# Comparison of real-world data (RWD) analysis on efficacy and post-progression outcomes with pembrolizumab plus chemo vs chemo alone in metastatic non-squamous non-small cell lung cancer with PD-L1 < 50%

**DOI:** 10.3389/fonc.2022.980765

**Published:** 2022-08-10

**Authors:** Ilaria Attili, Carmine Valenza, Celeste Santoro, Gabriele Antonarelli, Pamela Trillo Aliaga, Ester Del Signore, Chiara Catania, Gianluca Spitaleri, Antonio Passaro, Filippo de Marinis

**Affiliations:** ^1^ Division of Thoracic Oncology, European Institute of Oncology IRCCS, Milan, Italy; ^2^ Division of New Drug Development, European Institute of Oncology IRCCS, Milan, Italy; ^3^ Università degli Studi di Milano, Department of Oncology and Hemato-Oncology, Milan, Italy

**Keywords:** NSCLC, immunotherapy, combination, chemotherapy, platinum-doublet, sequential treatment

## Abstract

**Background:**

Following the introduction of immunotherapy (IO) in the first-line (1L) treatment in patients with non-small cell lung cancer (NSCLC) without sensitizing EGFR/ALK mutations, increasing real-world data depict how difficult it is to replicate data from clinical trials to clinical practice, with high rates of early treatment failure. In the context of chemo-IO, our study aims to compare platinum-pemetrexed-pembrolizumab combination to platinum-doublet alone in patients with low PD-L1 (<50%).

**Methods:**

We retrospectively collected medical records from patients with stage IV non-squamous NSCLC with PD-L1<50%, consecutively treated at our Centre from 2016 to 2021. Patients were grouped according to 1L treatment received: chemo-IO (group A) or platinum-doublet (group B). Survival outcomes were analyzed and compared among the two groups.

**Results:**

Overall, 105 patients were included: 49 in group A and 56 in group B. At data cut-off, median follow-up was 12.4 and 34.8 months, with 32/49 and 52/56 events for progression-free survival (PFS) and 21/49 and 29/56 events for overall survival (OS), respectively. No difference in PFS was observed between group B and group A (6.6 versus 8 months, HR 1.12, 95%CI 0.57-1.40). Patients receiving 1L platinum-doublet had significantly longer OS compared to those receiving chemo-IO (median OS 23.8 vs 14.9 months, HR 0.47, 95% CI 1.15- 3.98, p=0.01). 12 month-OS was 58% (95% CI 44-76%) in group A and 78% (95% CI 68-91%) in group B (p=0.040). Subgroup analysis identified KRAS G12C mutation as potentially affecting PFS in patients receiving chemo-IO (HR 0.29, 95% CI 0-10-0.91). The OS benefit of platinum-doublet was consistent across subgroups, with particular benefit in female sex, liver or pleural metastases, PD-L1 negative. Overall, only 46.9% of patients with progression received subsequent treatment in group A (15/32), compared to 86.5% in group B (45/52, all receiving 2L IO), with no difference in PFS to 2L (group A 3.7months, group B 4.1months, p=0.3).

**Conclusions:**

Despite small study population and differential follow-up, our study demonstrates that sequential use of 1L platinum-doublet and 2L IO is not inferior to 1L chemo-IO in non-squamous NSCLC with PD-L1<50%. In addition, we identified subgroups who might benefit differentially from the two approaches.

## Introduction

Immunotherapy (IO), in the form of immune checkpoint inhibitors (ICI), is the standard first-line (1L) therapy in patients affected by non-small cell lung cancer (NSCLC) without epidermal-growth factor receptor (EGFR) or anaplastic lymphoma kinase (ALK) gene alterations. In detail, ICI is currently approved by the Food and Drug Administration (FDA) and the European Medical Agency (EMA) as single-agent for NSCLC with high (≥50%) expression of programmed death ligand 1 (PD-L1), and in combination with platinum-based chemotherapy regardless of PD-L1 expression (<50%) (1, 2).

ICIs first showed clinical activity in the patient population with platinum-pretreated NSCLC (3–7). In this setting, anti-PD-1 immunotherapy with either nivolumab or pembrolizumab both resulted in prolonged overall survival (OS) compared to docetaxel, with hazard ratio (HR) for death of 0.59 (0.44-0.79, 95% CI; p<0.001) and of 0.73 (0.59-0.89, 96% CI; p=0.002) for nivolumab in squamous (SCC) and non-SCC histology, respectively, and of 0.70 (0.61-0.80, 95% CI) for pembrolizumab (3, 4, 6, 8). In addition, immunotherapy with the anti-PD-L1 agent atezolizumab also prolonged OS in the OAK clinical trial, with HR for death of 0.73 (0.62-0.87, 95% CI; p=0.0003) (5), while avelumab failed to improve OS in the JAVELIN Lung 200 trial, with an HR of 0.90 (0.72-1.12, 96% CI; p=0.16) (7).

**Figure 1:**
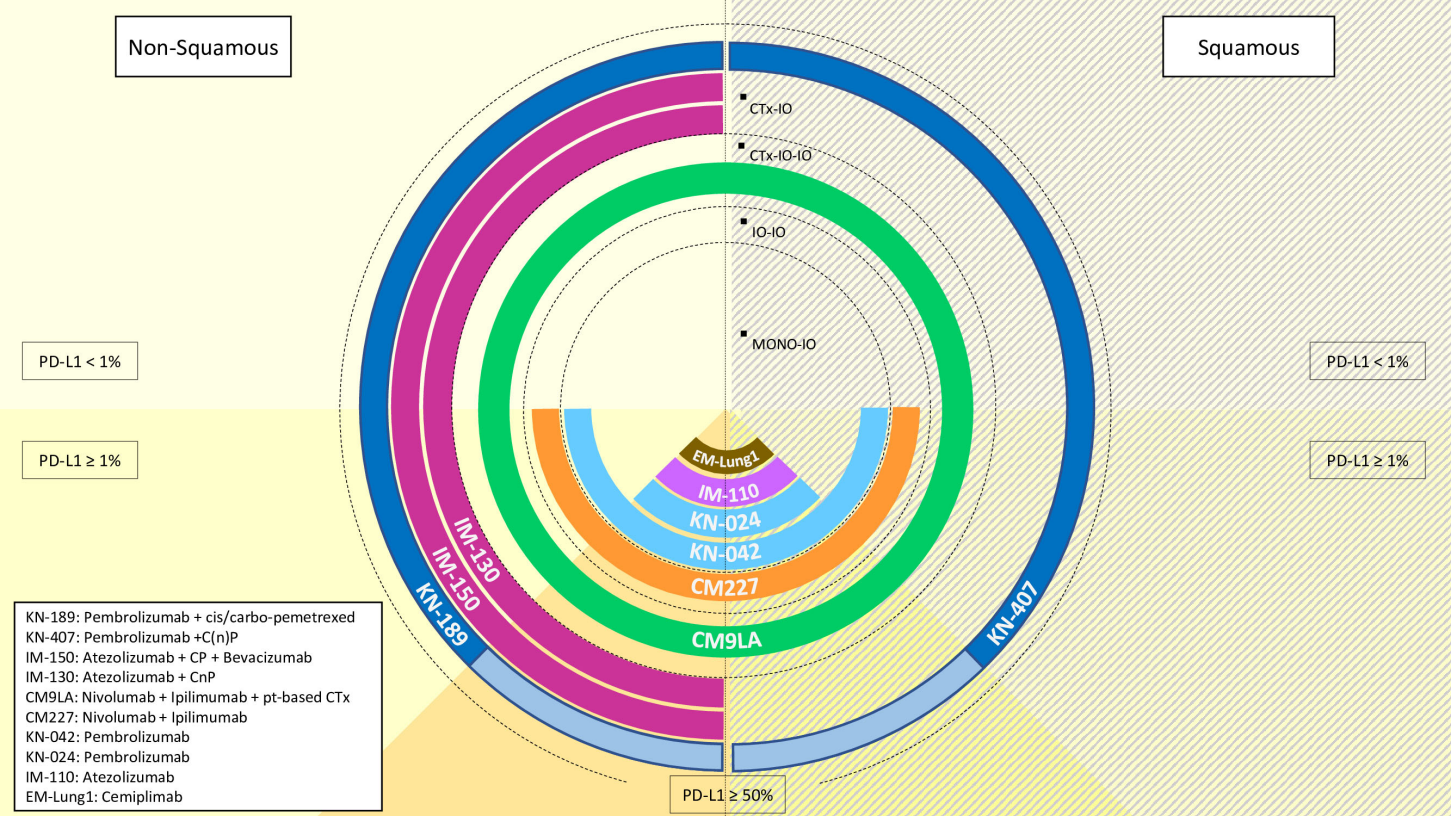
Overview of the available treatment options with immunotherapy (IO) in metastatic non-small cell lung cancer, according to histology (non-squamous: left-hand side, squamous: right hand side) and PD-L1 status (<1%: half-top side, ≥1%: half-bottom side). Treatments are codified as per their referral clinical trial name for approval. IO: immunotherapyCTx: chemotherapyC: carboplatinn: nabP: paclitaxelpt: platinum.

Building on these achievements, ICI had subsequently been tested from the pre-treated to the upfront, 1L, setting (9–14). In the KEYNOTE-189 clinical trial, pembrolizumab plus platinum-based doublet chemotherapy prolonged median OS and progression-free survival (PFS) from 10.7 (8.7-13.6, 95% CI) to 22.0 months (19.5-25.2, 95% CI), and from 4.9 (4.7-5.5, 95% CI) to 9.0 months (8.1-9.9, 95% CI), respectively (9). In the IMpower130 clinical trial, atezolizumab lengthened both median PFS from 5.5 (4.4-5.9, 95% CI) to 7 months (6.2-7.3, 95% CI) as well as median OS from 13.9 (12.0-18.7, 95% CI) to 18.6 months (16.0-21-2, 95% CI) in the non-SCC NSCLC setting (13). Moreover, also the addition of atezolizumab to the bevacizumab-carboplatin-paclitaxel regimen improved OS from 14.7 to 19.0 months with an HR for death of 0.80 (0.67-0.95, 95% CI) in patients with non-SCC NSCLC in the IMpower150 clinical trial (11, 12). Of note, also the front-line IO combination nivolumab plus ipilimumab (anti-cytotoxic T lymphocyte antigen-4, anti-CTLA-4) added to two cycles of platinum-based chemotherapy resulted in prolonged median OS from 10.9 (9.5-12.6, 95% CI) to 15.6 months (13.9-20.0, 95% CI) with and HR for death of 0.66 (0.55-0.80, 95% CI) compared to four cycles of chemotherapy alone in the CheckMate 9LA clinical trial (14) (**Figure 1**).

Notably, in the aforementioned clinical trials the beneficial effect of ICI on median OS was consistent regardless of PD-L1 expression, largely because of the high percentage of patients in the control arms being treated with an IO upon progression. Instead, both the overall response rate (ORR) as well as the duration of response (DOR) were more positively impacted by pembrolizumab or atezolizumab in patients with a high PD-L1 (≥50%) compared to low (<50%) expression (10–13). Moreover, clinical trials investigating the role of front-line immunotherapy in patients with high PD-L1 expression (≥50%), such as the KEYNOTE-024 and the EMPOWER-Lung 1, showed the superiority of ICI monotherapy compared to platinum-based chemotherapy (15, 16). In detail, sub-groups of patients benefitting the most from ICI were good performance status (PS 0-1), reduced number of metastatic sites (0-1) as well as bone or liver involvement (17). Novel predictive biomarkers are currently being investigated in this context also prospectively, such as for the blood-derived tumor mutational burden ≥16 in the B-F1RST clinical trial (NCT02848651) (18).

A pooled analysis from randomized controlled trials with IO as mono-therapy or chemo-IO was performed by FDA, showing advantage of chemo-IO over IO alone in patients with PD-L1 expression of 1-49% (19). The same approach was applied to investigate outcomes in patients with high PD-L1 levels, showing similar outcomes with the two IO-based regimens, and this appears confirmed from a large real-world study in non-squamous histology (20, 21).

Importantly, real-world data depict how difficult it is to replicate results obtained from pivotal clinical trials into clinical practice especially in patients with low PD-L1 expression (<50%) (22–24). This is due to several factors, among which high rates of early treatment failure, patients’ heterogeneity, as well as the lack of definitive biomarkers predictive of IO response.

In this scenario, it is important to collect complete clinical and pathological data alongside biological information regarding patients with advanced NSCLC being treated with upfront chemo-immunotherapy to better clarify which subgroup of patients would benefit the most from this approach as well as to identify therapeutic alternatives for patients who do not advantage from this combinatorial treatment strategy (17, 25, 26). In this context, real-world comparative studies between chemo-IO and IO alone or chemotherapy alone are lacking due to different reasons: IO monotherapy is preferred in patients with high PD-L1 expression and chemo-IO is mostly adopted in PD-L1 <50%, whereas chemotherapy alone is currently no more considered as standard treatment option. However, the possibility to adopt a sequential treatment and replicate long-term survival outcomes reported with ICI in pretreated patients remains highly attractive in this setting.

Hence, our study focuses on the retrospective assessment of patients with NSCLC and low PD-L1 expression (<50%) being treated either with upfront chemo-immunotherapy combination (pembrolizumab plus platinum salt plus pemetrexed) versus chemotherapy alone (platinum-based doublet chemotherapy) followed by second-line immunotherapy upon disease recurrence.

## Materials and methods

### Patients

We conducted a retrospective real-world study on patients with stage IV non-squamous NSCLC and PD-L1 <50%, consecutively treated at our Centre to compare the outcomes of first-line chemo-immunotherapy combination (PPP: carboplatin or cisplatin plus pemetrexed plus pembrolizumab) with the previous standard of platinum-doublet chemotherapy only approach. Based on the time of first approval of mono-immunotherapy in pretreated patients in Italy, we selected the study period from 2016 to 2021, defining two study populations: patients treated after December 2019 (approval data in Italy of PPP for non-squamous NSCLC with PD-L1 <50%) received PPP chemo-IO combination as first-line treatment (group A), whereas patients treated before that date received first-line platinum-doublet chemotherapy alone (group B).

Medical records of patients included in the study were reviewed to collect clinical information, including demographics, baseline clinical features, tumor, and treatment-related data. Only patients with adequate follow-up information, including disease status or death at database lock, and complete clinical records were considered for study analysis.

All the study procedures were carried out by the general authorization to process personal data for scientific research purposes from “The Italian Data Protection Authority” (http://www.garanteprivacy.it/web/guest/home/docweb/-/docwebdisplay/export/2485392, accessed on 10 April 2022). All information regarding subjects was managed using anonymous numerical codes and handled in compliance with the Declaration of Helsinki. According to the aforementioned national guidelines, the study did not require an Ethical Committee approval since it did not affect the clinical management of the involved patients. Informed consent was obtained from all subjects involved in the study.

### Study endpoints

The primary endpoint was to compare OS of patients with non-squamous NSCLC receiving first-line chemo-immunotherapy combination (group A) with OS in those receiving first-line platinum-doublet alone (group B) in the real-world setting.

Secondary endpoints were OS in patients receiving a second-line treatment, PFS, and safety. PFS to second-line treatment and rate of patients who receive second-line treatment between the two groups were exploratory endpoints.

### Statistical analysis

Variables were presented using the median value for continuous variables and percentages (numbers) for categorical variables. Mann-Whitney test was used to compare continuous variables, whereas two-sided chi-squared or fisher-exact test were used to compare categorical variables, as appropriate.

OS was defined as the time between the start of first-line treatment and the occurrence of death from any cause. PFS was defined as the time between the start of first-line treatment and progression or death from any cause. In patients who received any second-line treatment, PFS2 was defined as the time between the start of second-line treatment and progression or death from any cause.

Median PFS, PFS2 and OS were estimated by using Kaplan–Meier methods. Median follow-up was calculated with the reverse Kaplan–Meier method. A z-test was used to compare 12-month OS rate between groups. The Cox regression model was used for subgroup analysis on survival outcomes, and data were presented as hazard ratios (HR) or odds ratios (OR) and their 95% confidence interval (CI), as appropriate.

Statistical significance level was set at p < 0.05 for all tests. All statistical analyses were performed with R Studio version 4.1.2.

## Results

### Patients and treatments

A total of 105 patients with advanced NSCLC and without known actionable gene alterations, consecutively treated at our Center in the study period, met the inclusion criteria for our study. Overall, 49 patients were treated after commercialization in Italy of pembrolizumab in combination with platinum-pemetrexed chemotherapy and received PPP regimen as their first-line treatment (group A). 56 patients treated before December 2019 received a first-line platinum-doublet chemotherapy alone (group B). All patients received molecular testing with local next-generation sequencing panels and were found to have *EGFR*/*ALK*/*ROS1*/*BRAF* wild-type tumors.

Overall, clinical characteristics were comparable between the two groups. Median age was 68 (44-79) in group A and 67 (45-76) in group B, with prevalence of male sex in 63.3% and 57.1%, respectively. All patients (100%) had non-squamous histology. Groups were balanced also in terms of smoking status, ECOG PS, presence or absence of comorbidities, and PD-L1 levels (<1% or 1-49%), as well as in the distribution of main metastatic sites (liver, pleura, brain). Of note, less patients had 2 or more metastatic sites in group B compared to group A (39.3% and 69.4%, respectively) (**Table 1**).

**Table 1:** Clinical characteristics of study population.

	A (n=49)	B (n=56)	P value^a^
Age, median (range)	68 (44-79)	67 (45-76)	0.49
Sex			
male, n (%) Female, n (%)	31 (63.3) 18 (36.7)	32 (57.1) 24 (42.8)	0.66
Smoking status			
Current Former Never unknown	15 (30.6) 29 (59.2) 3 (6.1) 2 (4.1)	13 (23.2) 30 (53.6) 7 (12.5) 6 (10.7)	0.37
Histology			
non-squamous, n (%)	49 (100)	49 (100)	1
ECOG PS			
0 1 ≥2	6 (12.2) 41 (83.7) 2 (4.1)	10 (17.8) 44 (78.6) 2 (3.6)	0.84
Major comorbidities			
Yes, n (%) No, n (%)	28 (57.1) 21 (42.9)	27 (48.2) 29 (51.8)	0.47
N mets			
0-1 ≥2	15 (30.6) 34 (69.4)	34 (60.7) 22 (39.3)	0.004
KRAS			
Mut G12C non-G12C WT	23 (46.9) 14 (28.6) 9 (18.3) 26 (53.1)	21 (37.5) 6 (10.7) 15 (26.8) 35 (62.5)	0.43
PD-L1			
0, n (%) 1-49%, n (%)	16 (32.7) 33 (67.3)	21 (37.5) 35 (62.5)	0.43
Brain			
Yes, n (%) No, n (%)	9 (18.4) 40 (81.6)	11 (19.6) 45 (80.4)	1
Liver			
Yes, n (%) No, n (%)	7 (14.3) 42 (85.7)	2 (3.6) 54 (96.4)	0.11
Pleura			
Yes, n (%) No, n (%)	19 (38.8) 30 (61.2)	16 (38.6) 40 (71.4)	0.37
Bone			
Yes, n (%) No, n (%)	22 (44.9) 27 (55.1)	13 (23.2) 43 (76.8)	0.03
Extra-thoracic M sites			
Yes, n (%) No, n (%)	33 (67.3) 16 (32.7)	29 (51.8) 27 (48.2)	0.16

^a.^Mann-whitney for continuous variables, chi-squared or fisher test for categorical variables.

At data cut-off, median follow-up was 12.4 in group A and 34.8 months in group B.

Most patients (96%) in group A received a combination of carboplatin with pemetrexed and pembrolizumab, with only 2 patients (4%) receiving cisplatin. Conversely, in group B, 26 (46.4%) patients received a cisplatin-based treatment. Furthermore, in group B, pemetrexed was administered only in 75% of patients. Median number of platinum-doublet chemotherapy cycles was 3 in group A versus 4 in group B (p=0.009). Median number of maintenance treatment cycles administered was 4 (range 0-31) versus 0 (range 0-25), respectively (p=0.04). No difference was observed in terms of carboplatin AUC doses between the two groups (**Supplementary Table 1**).

### Survival analysis

**Figure 2:**
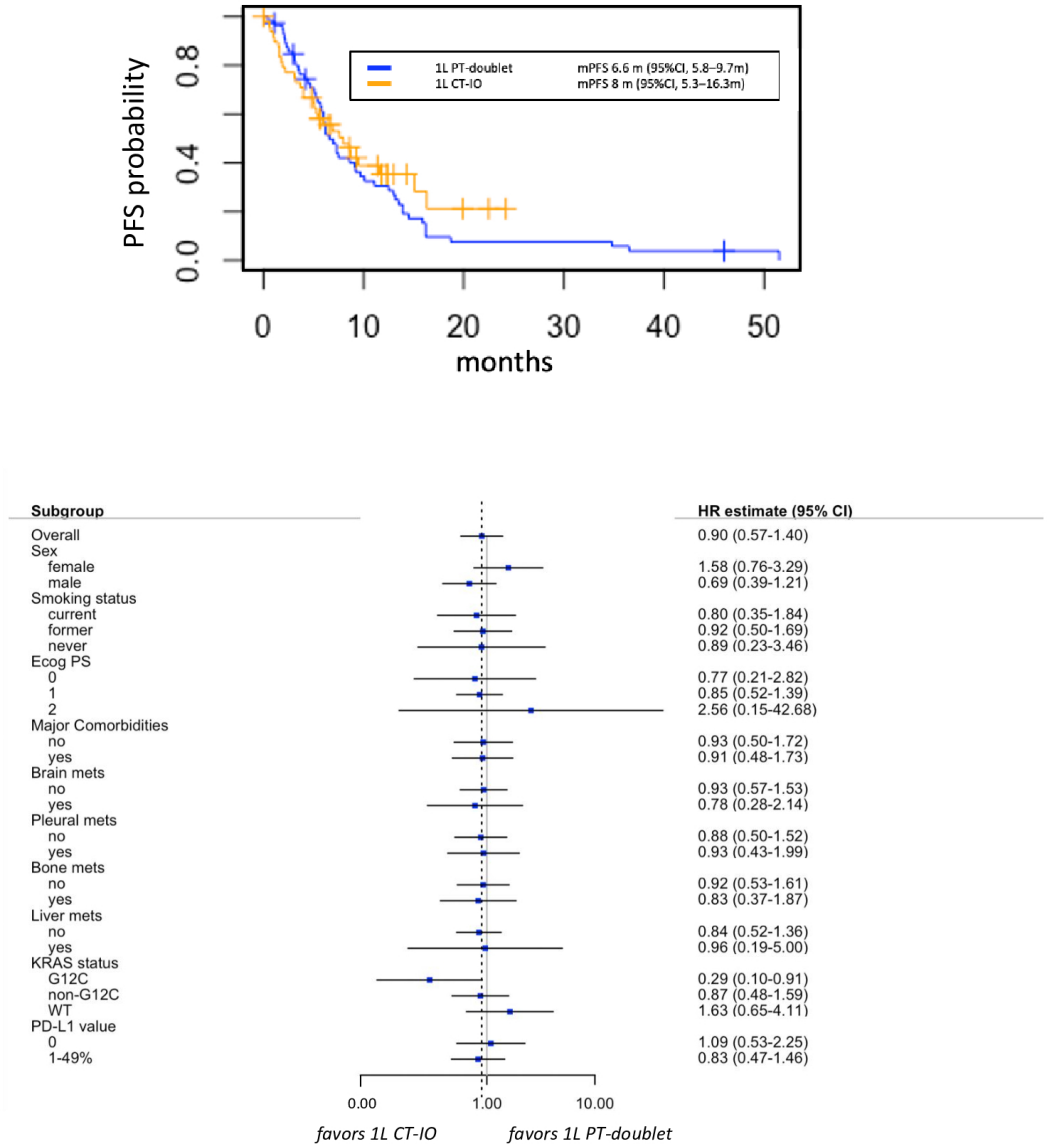
Progression free survival (PFS) in group A versus group B (upper panel), with associate subgroup analysis (lower panel). 1L, first line; CT-IO, chemo-immunotherapy; PT, platinum.

At data cut-off (March 2022), 32 out of 49 (65.3%) and 52 out of 56 (92.9%) events for PFS occurred in group A and group B, respectively. Median PFS was 8 months (95% CI 5.3-15.1 months) in the chemo-IO group versus 6.6 months (95% CI 5.8-9.7 months) in the chemotherapy alone group (HR 1.12, 95% CI 0.57-1.40, p=0.6). The absence of differential benefit between the two treatment groups was consistent across the subgroups evaluated, including smoking history, metastatic sites and PD-L1 levels (**Figure 2**). Of note, the presence of KRAS G12C mutation seemed to predict greater PFS benefit with chemo-IO combination compared to chemotherapy alone (HR 0.29, 95% CI 0.10-0.91), however this signal should be further investigated in larger cohorts.

Overall, 21 out of 49 (42.9%) and 29 out of 56 (51.8%) events had occurred for OS in group A and group B, respectively, displaying substantially similar data maturity despite the different follow-up time.

**Figure 3:**
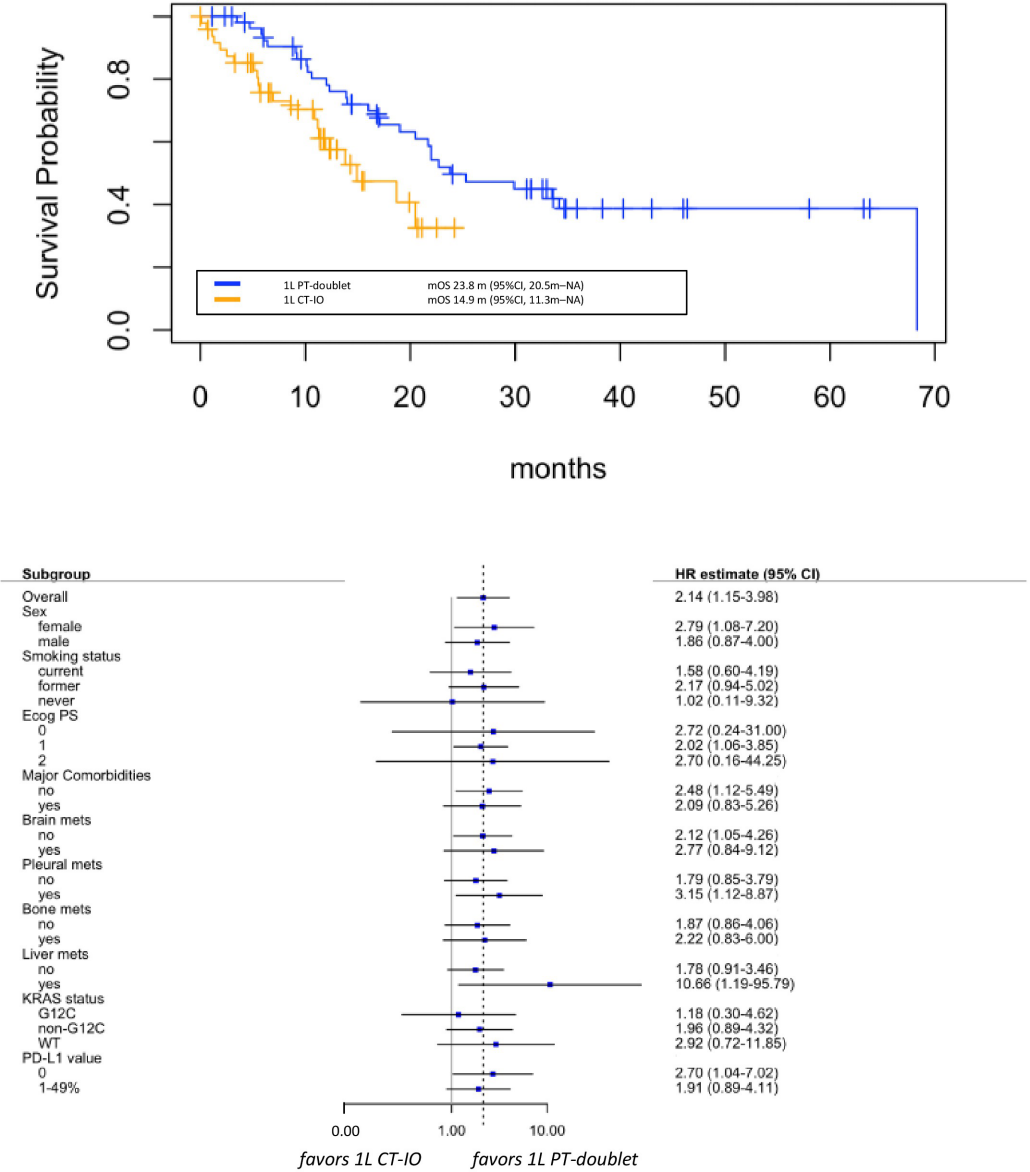
Overall survival (OS) in group A versus group B (upper panel), with associate subgroup analysis (lower panel). 1L: first lineCT-IO: chemo-immunotherapyPT: platinum.

Median OS was 23.8 months (95% CI 20.5 months-NA) in patients receiving platinum-doublet alone and 14.9 months (95% CI 11.3 months-NA) in those treated with the chemo-IO combination, with statistically significant death risk reduction in the chemotherapy alone group (HR 0.47, 95% CI 1.15- 3.98, p=0.01) (**Figure 3**).

Considering the different follow-up period in group A and group B, we also assessed 12-month OS rates to provide a more reliable comparison between the two treatment groups. At one year, 17 deaths (35%) occurred in patients receiving first-line PPP, whereas only 11 events (21%) occurred among those who were treated with first-line platinum-doublet alone. The 12 month-OS rate was 58% (95% CI 44-76%) in patients in the combination treatment and 78% (95% CI 68-91%) in the chemotherapy group, confirming statistically significant difference favoring the first-line platinum-doublet alone treatment (p=0.040).

The benefit of the first-line chemotherapy alone was observed across all subgroups that were analyzed, with no statistically significant p interaction tests within subgroup categories (**Figure 3**). However, although very small numbers, signals of greater magnitude of benefit were observed in patients with female sex (HR 2.79, 95% CI 1.08-7.20), presence of liver (HR 10.66, 95% CI 1.19-95.79) or pleural metastases (HR 3.15, 95% CI 1.12-8.87), or absent PD-L1 expression (HR 2.07, 95% CI 1.04-7.02).

### Second-line treatment evaluation

We further investigated how the first-line approach could affect the subsequent treatment line in each group. Among patients progressing to first-line chemo-IO combination (n=32), only 15 (46.9%) received second-line treatment (10 docetaxel, 4 clinical trials, 1 *RET* inhibitor in patient with *RET* rearrangement). Of note, 12 patients (40%) died and could not receive any subsequent treatment.

Conversely, 45 out of 52 (86.5%) patients who progressed on first-line platinum-doublet alone received a second-line treatment, all with an immune checkpoint inhibitor monotherapy. Only four patients (7.7%) could not receive a second-line therapy because of worsening clinical conditions and death.

We further analyzed survival in the second-line treatment population. Overall, 9 out of 15 (60%) and 36 out of 45 (80%) progression events occurred in the second line setting in the two groups, respectively.

In the second-line population, median OS from the start of first-line treatment was 14.9 months (95% CI 11.9 months - NA) in group A, versus 25.3 months (95% CI 21.7 months-NA) in group B, confirming reduced survival in patients receiving the combination treatment of platinum-doublet and immune checkpoint inhibitor compared to the sequential approach (HR 2.23, 95% CI 0.90-5.53, p = 0.07).

We observed no difference in PFS to second-line treatment between the two groups: median PFS2 was 3.7 months (95% CI 2.1 months-NA) in the previously PPP treated group A versus 4.1 months (95% CI 3.2-11.1 months) in group B (p=0.3) (**Supplementary Figure 1**).

### Safety

Adverse events (AEs) to the first-line treatment were collected and analysed for the entire study cohort. Overall, patients in group A had 46.7% incidence of grade 3 or higher AEs, whereas the incidence was 17.9% in group B (odds ratio OR 4.31, 95% CI 1.797-10.99). Immune-related AEs (irAEs) occurred more frequently in patients receiving the combination with ICI: any-grade irAEs were 17 (34.7%) in group A, compared to 7 (12.5%) in group B (OR 3.63, 95% CI 1.38-10.54). Grade≥3 irAEs were 9 (18.3%) and 2 (3.6%), respectively (**Supplementary Table 2**). Of note, similar discontinuation rate due to adverse events were observed in the two groups (20.4% and 19.6%, respectively).

The most common AEs of grade ≥3 were pneumonitis, occurring in 5 patients (10.1%) in group A and 1 patient (1.8%) in group B, colitis, pulmonary embolism and neutropenia (**Supplementary Table 3**).

## Discussion

The use of ICIs, dramatically chagend the treatment scenario of patients affected by NSCLC, both in first-line and pretreated setting. In non-squamous population, unselected for PD-L1, 3-year OS rate with pembrolizumab plus pemetrexed-platinum was 31.3% vs 17.4% with placebo plus pemetrexed-platinum (10).

In the pre-treated setting, five-year pooled OS rates from the CheckMate 017-057 trials were 13.4% versus 2.6%, respectively, 18.3% (95% CI, 13.0 to 24.2) versus 3.4% (95% CI, 1.4 to 6.8) in patients with PD-L1 expression ≥ 1% and 8.0% (95% CI, 4.4 to 13.0) versus 2.0% (95% CI, 0.5 to 5.3) in those with PD-L1 expression < 1%. Median OS in non-squamous population was 12.2 (95% CI, 9.7 to 15.1) vs 9.5 (95% CI, 8.1 to 10.7) months (27). 5-year OS rates for pembrolizumab versus docetaxel in the KEYNOTE-010 trial (squamous and non-squamous histology) were 25.0% versus 8.2% in patients with PD-L1 ≥50% and 15.6% versus 6.5% with PD-L1 ≥1% (8).

As discussed above, reports are available confirming that the outcomes of first-line chemo-immunotherapy are inferior to those obtained in the real-life (22, 23). Hence, our real-world study aimed at assessing whether the possibility to sequence chemotherapy and immunotherapy would be beneficial instead of the standard chemo-immunotherapy approach, focusing on the subgroup of patients with non-squamous histology and PD-L1 <50%.

In this light, we cannot adequately compare our results with those of clinical trials that are pooled both for histology and PD-L1, with no data available for the specific subgroup of non-squamous with PD-L1 0-49%. Similarly, data in the KEYNOTE-189 trial are presented separately for PD-L1 negative and PD-L1 1-49% (3-year OS rate of the combination vs platinum-doublet was 28.3% vs 17.2% and 23.3% vs 5.3%, respectively) (10).

Our study has limitations related to its retrospective design and differential follow-up of the two cohorts of patients. However, according to the current standard of care, platinum-doublet chemotherapy alone is not indicated in clinical practice as first-line treatment. Indeed, chemo-immunotherapy demonstrated to be superior to chemotherapy alone in clinical trials, and a prospective evaluation would be not feasible in the absence of at least hypothesis-generating data as those that we are reporting. To limit the bias related to the differential follow up, we assessed the 12-month OS rate for each group of patients. Of note, the advantage of the chemotherapy alone approach is maintained in this evaluation. Of course, we acknowledge that a propensity score matching analysis would add value in the retrospective setting to further unbias the overall findings, however, due to the limited number of this study, we could not perform such kind of analysis.

Despite the mono-centric nature of our work and the related study samples, to our knowledge this represents the first reported work comparing the two regimens in the front-line setting of patients with non-squamous NSCLC and PD-L1<50%, with focus also on results with subsequent treatments. Of great interest, we observed that the majority (86.5%) of patients who received chemotherapy alone as first-line treatment could effectively receive second-line immunotherapy (the rate of effective cross-over in the KEYNOTE-189 was 53.9% in the control arm). Conversely, 40% of patients who received first-line chemo-immunotherapy could not receive any subsequent treatment because of worsening of clinical conditions and death.

Of note, in our study, we also focused on results with second-line treatments, limiting to those patients who could receive them. Therefore, these results cannot be compared to PFS-2 results of the KEYNOTE-189 trial (17 vs 9 months from randomization to progression to the second-line treatment)^10^.

When observing such real-world worse outcomes with chemo-immunotherapy with respect to clinical trials, we hypothesized a role of personalized dosages of combined chemotherapy administered in combination with pembrolizumab. However, even though higher number of patients received cisplatin in the chemotherapy alone group, no differences were observed in the median AUC of carboplatin in the two groups. Of note, due to absence of differential impact on survival with cisplatin or carboplatin in the KEYNOTE-189 trial, cisplatin was not routinely adopted in the chemo-immunotherapy arm at our centre. Hence, the choice of carboplatin instead of cisplatin do not reflect a difference in the clinical status of patients treated in our study. Patients in the chemo-immunotherapy arm were more likely to receive less cycles of treatment (median 3 vs 4) in the induction phase, and this might be related to a worse tolerability of the combination. Indeed, despite the limits of retrospective data collection of AEs, grade≥3 AEs were more than doubled in the combination group compared to the chemotherapy group (46.7% vs 17.9%), with nearly 20% of grade≥3 irAEs.

As expected, maintenance treatment was more prolonged in group A, due to the absence of maintenance schedule in non-pemetrexed based regimens in group B and to the possibility to prolong pembrolizumab treatment alone.

The results of our study are not expected to change the standard approach in first-line treatment of non-squamous NSCLC, however they are highly hypothesis generating for different aspects.

As first, they are confirming that real-life population has different outcomes compared to clinical trial population, and this should be always considered by clinicians when starting a chemo-immunotherapy regimen.

Second aspect is the attracting possibility of obtaining an overperformance from the front-line platinum-doublet alone through the possibility to treat virtually all patients with second-line immunotherapy, therefore reaching long-term results similar to those observed in clinical trials conducted in the pretreated setting.

In our study, we found the presence of KRAS G12C mutation predicting greater PFS benefit with chemo-IO combination compared to chemotherapy alone, confirming a role of ICI in KRAS-mutant disease (28). Of intriguing impact, we observed OS advantage with first-line platinum-doublet alone across subgroups, but we identified female sex, presence of liver or pleural metastases and absent PD-L1 expression as categories with greater magnitude of benefit with front-line ICI-sparing approach. Differently from observations in the comparison between mono-IO and chemo-IO, we observed no impact of smoking status on treatment results between chemotherapy and chemo-IO.

Due to the limited number of patients, all these signals should be further investigated in larger cohorts.

In conclusion, we are first reporting on comparison between standard chemo-immunotherapy with platinum-pemetrexed-pembrolizumab versus platinum-doublet alone front-line approach in patients with non-squamous NSCLC and PD-L1<50%. Results are interesting in the light of potentially define patients who might have detrimental results from a combination treatment both in terms of survival and AEs. According to our results, future research should be addressed to identify and select patients who might be better candidate to an ideally sequential approach of de-escalated treatment with platinum-based chemotherapy followed by immunotherapy at disease progression. Also, further investigation should be conducted in the real life to assess whether the adoption of other chemo-immunotherapy regimens with less chemotherapy (e.g., only 2 cycles of platinum-based chemotherapy) (29) could be beneficial in the real-world population in terms of clinical performance and safety, with respect to platinum-pemetrexed-pembrolizumab and to chemotherapy alone.

## Data availability statement

The raw data supporting the conclusions of this article will be made available by the authors, without undue reservation.

## Ethics statement

Ethical review and approval was not required for the study on human participants in accordance with the local legislation and institutional requirements. The patients/participants provided their written informed consent to participate in this study.

## Author contributions

Conceptualization: IA and FdM; methodology and data analysis: IA; data collection: IA, CV, CS, GA; patients: IA, PTA, ES, GS, CC, AP, FdM; writing—original draft preparation: IA, GA, and AP; writing—review and editing: all authors; visualization: IA; supervision: FdM. All authors have read and agreed to the published version of the manuscript.

## Funding

This work was partially supported by Associazione Italiana di Oncologia Toracica (AIOT) (no grant number) and the Italian Ministry of Health with “Ricerca Corrente”, “5 x 1000”.

## Conflict of interest

AP reports personal fees, as speaker bureau or advisor, for AstraZeneca, Agilent/Dako, Boehringer Ingelheim, Bristol-Myers Squibb, Eli-Lilly, Merck Sharp & Dohme, Janssen, Novartis, Pfizer and Roche Genentech, outside the submitted work; FdM has served in a consultant/advisory role for Astra Zeneca, Boehringer Ingelheim, Bristol-Myers Squibb, Celgene, Merck Sharp & Dohme, Novartis, Roche Genentech, Takeda and Pfizer, outside the submitted work.

The remaining authors declare that the research was conducted in the absence of any commercial or financial relationships that could be construed as a potential conflict of interest.

## Publisher’s note

All claims expressed in this article are solely those of the authors and do not necessarily represent those of their affiliated organizations, or those of the publisher, the editors and the reviewers. Any product that may be evaluated in this article, or claim that may be made by its manufacturer, is not guaranteed or endorsed by the publisher.
