# Supplementary material for: Comparison of real-world data (RWD) analysis on efficacy and post-progression outcomes with pembrolizumab plus chemo vs chemo alone in metastatic non-squamous non-small cell lung cancer with PD-L1 < 50%
Source: Front Oncol. 2022 Aug 10;12:980765. doi: 10.3389/fonc.2022.980765 (PMC9399686; doi:10.3389/fonc.2022.980765)
Supplement: Supplementary file 1 [file DataSheet_1.docx]

Supplementary Material

# Supplementary Tables

**Supplementary Table 1. First-line treatment details in study population**

|  | Group A | Group B |  |
| --- | --- | --- | --- |
| 1L (n) | Carboplatin+pemetrexed+pembrolizumab (46)  Cisplatin+pemetrexed+pembrolizumab (2) | Carboplatin+pemetrexed (22)  Cisplatin+pemetrexed (20)  Carboplatin+Gemcitabine (8)  Cisplatin+Gemcitabine (5)  Cisplatin+Paclitaxel (1) |  |
| Median cycles (range)  Platinum-doublet  Maintenance | 7 (1-35) 3 (1-4) 4 (0-31) | 4.5 (1-29)  4 (1-6)  0 (0-25) | p=0.48  p=0.009  p=0.04 |
| Median carboplatin AUC (range) | 4 (3-6) | 4 (3-5) | p=0.17 |

**Supplementary Table 2. Adverse events according to treatment group**

|  | Group A (n=49) | Group B (n=56) | OR (95% CI) |
| --- | --- | --- | --- |
| Grade ≥3 adverse events | 23 (46.7%) | 10 (17.9%) | 4.31 (1.797-10.99) |
| Any-grade immune related adverse events  Adverse events leading to treatment discontinuation | 17 (34.7%)  10 (20.4%) | 7 (12.5%)  11 (19.6%) | 3.63 (1.38-10.54) |
| Grade ≥3 immune related adverse events | 9 (18.3%) | 2 (3.6%) | 1.37 (0.16-7.33) |

**Supplementary Table 3. Distribution of grade ≥3 adverse events according to treatment group**

|  | Group A (n=49) | Group B (n=56) |
| --- | --- | --- |
| pneumonitis | 5 (10.2%) | 1 (1.8%) |
| colitis | 2 (4.1%) | - |
| Pulmonary embolism | 3 (6.1%) | - |
| cholangitis | 1 (2%) | - |
| asthenia | 2 (4.1%) | - |
| neuropathy | 2 (4.1%) | - |
| Pulmonary fibrosis | 1 (2%) | - |
| neutropenia | 3 (6.1%) | 2 (3.6%) |
| thrombocytopenia | 2 (4.1%) | 2 (3.6%) |
| anemia | 2 (4.1%) | 3 (5.4%) |
| Impaired renal function | 2 (4.1%) | 2 (3.6%) |
| diarrhea | 2 (4.1%) | 1 (1.8%) |
| erysipelas | 2 (4.1%) | 1 (1.8%) |
| Increased transaminases | 2 (4.1%) | 1 (1.8%) |

## Supplementary Figures

**
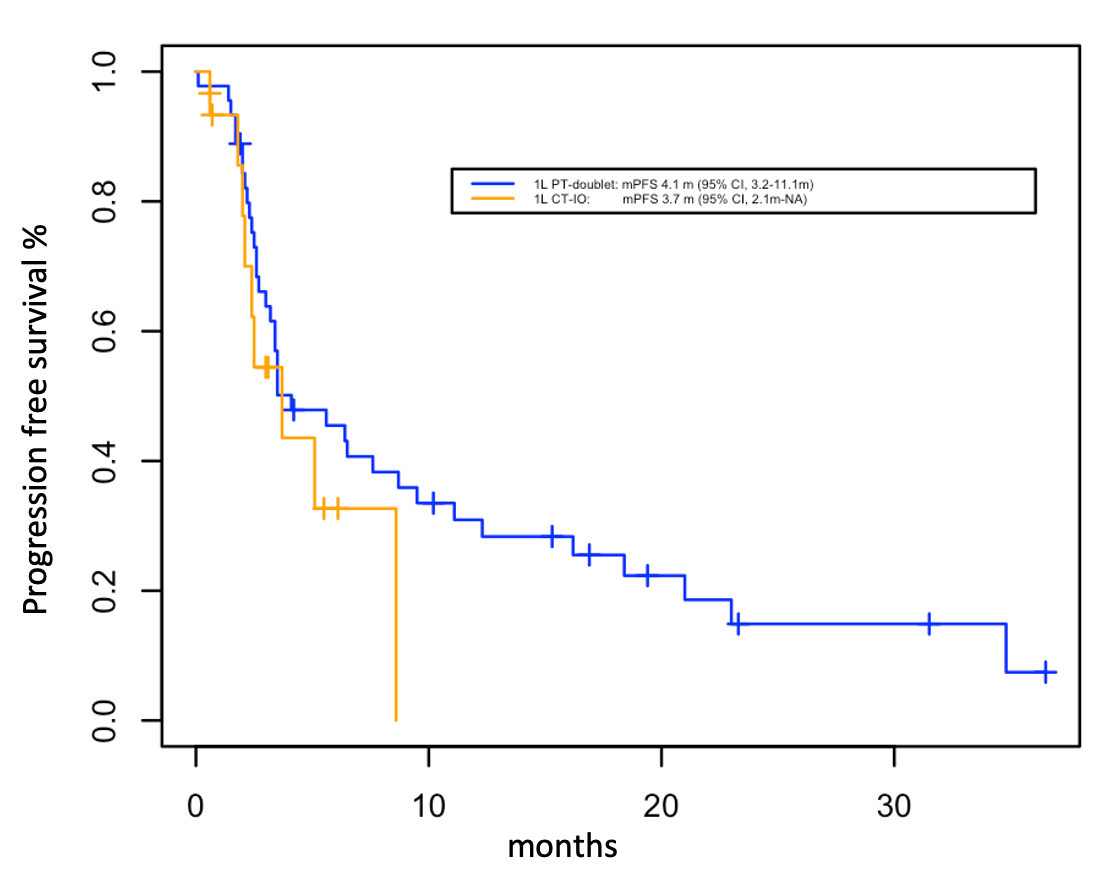
**

**Supplementary Figure 1.** Progression free survival (PFS) to second-line treatment between the two groups: median PFS2 was 3.7 months (95% CI 2.1 months-NA) in group A (previously treated with platinum-pemetrexed-pembrolizumab) versus 4.1 months (95% CI 3.2-11.1 months) in group B (previously treated with platinum-doublet chemotherapy alone) (p=0.3)
